# Unmasking the “siltation-ınduced local tidal effect”: the lost art of bilateral blood pressure measurement in subclavian steal syndrome

**DOI:** 10.1016/j.bjorl.2026.101932

**Published:** 2026-10-14

**Authors:** Muhammed Emre Güleşir

**Affiliations:** Cardiology Clinic, Bolu Izzet Baysal State Hospital, Bolu, Turkey

Dear Editor,

I read with great interest the recent comprehensive review by Lima Neto et al.^1^ published in your valued journal. The authors elegantly categorize vertebrobasilar hypoflow conditions into “siltation effects” (structural flow reduction) and “tidal effects” (global perfusion impairment). This didactic approach supplies a highly applicable framework for clinical reasoning.

While the authors accurately classify Subclavian Steal Syndrome (SSS) as a classic siltation effect, I wish to expand on this concept by introducing a “siltation-induced local tidal effect”, notably in the context of the rare right-sided SSS.

In SSS, the structural obstruction (siltation) is not merely a localized blockade; when severe, it drastically lowers distal pressure, thereby reversing ipsilateral vertebral flow. This creates an isolated, retrograde hemodynamic “tide” (the steal phenomenon) that mimics the systemic tidal effects described by the authors, yet is entirely driven by proximal siltation. Conceptually, this positions severe SSS as a distinct entity within the authors' classification, appearing as an “overlapping condition”.

We recently demonstrated this complex overlapping pathophysiology in a 68-year-old male suffering from medically refractory vertigo.^2^ As Lima Neto et al. astutely note, only a small fraction of SSS cases become symptomatic, and the magnitude of the inter-arm blood pressure difference is the most important predictor of this symptomatic presentation.^1^ Our case perfectly demonstrates this principle: the diagnostic turning point was not a high-level neuroimaging modality, but a waning clinical art: discovering a staggering 40 mmHg systolic blood pressure gradient between his upper extremities. Subsequent angiography confirmed severe proximal right subclavian artery stenosis (the siltation) causing profound retrograde steal from the right vertebral artery (the local tide) (Fig. 1). Following percutaneous transluminal angioplasty and the deployment of a balloon-expandable stent, the patient achieved complete anatomical resolution and an instant cessation of his long-standing vertigo.

**Fig. 1:**
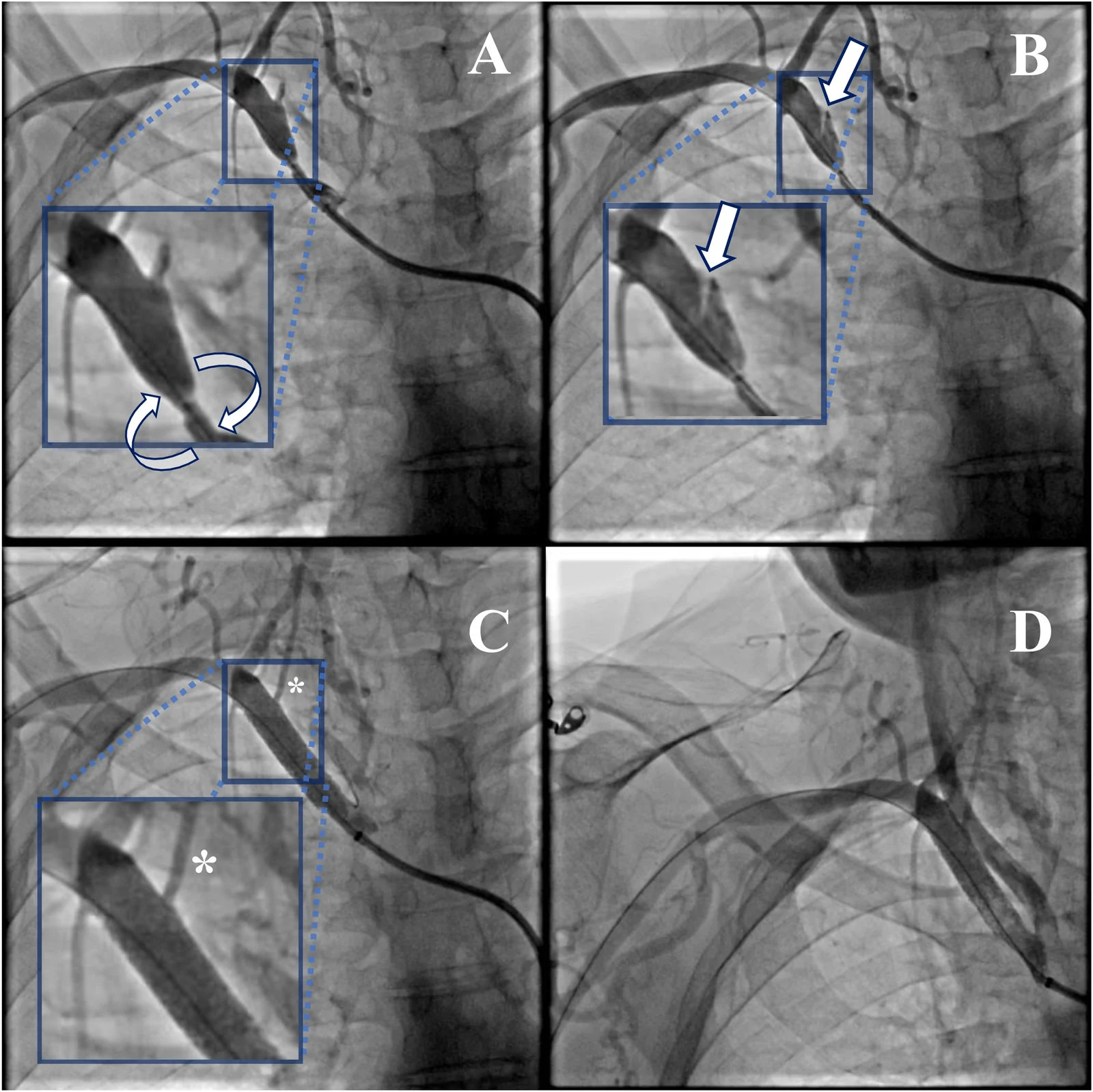
Treatment of right subclavian steal syndrome. (A) Proximal right subclavian stenosis, with a magnified inset showing curved arrows indicating severe narrowing (the “siltation”). (B) Retrograde filling from the right vertebral artery confirms the steal phenomenon, indicated by straight arrows in both the main image and inset. (C) Deployment of a 9 × 31 mm stent across the stenosis. The asterisk (*) marks the right vertebral artery, indicating where physiological antegrade flow is restored after stenosis resolution. (D) Final control angiography demonstrates complete anatomical resolution, restored antegrade flow, and elimination of steal. (Adapted from Gülesir et al.^2^ Available under the Creative Commons Attribution 4.0 International License).

Therapeutic success in vertebrobasilar hypoflow relies heavily on clinical suspicion. A systolic blood pressure difference of >15–20 mmHg between the arms remains the most sensitive clinical indicator for this phenomenon.

In conclusion, I highly commend Lima Neto and colleagues for their excellent mechanistic framework. However, I strongly advocate that any modern diagnostic algorithm for vertebrobasilar hypoflow must explicitly mandate routine bilateral blood pressure monitoring in the initial triage phase. A simple sphygmomanometer remains an essential instrument for unmasking complex, siltation-induced tidal effects such as right subclavian steal syndrome.

## Declaration of Generative AI and AI-assisted technologies in the writing process

During the preparation of this work, the author used Gemini in order to polish the English language. After using this tool/service, the author reviewed and edited the content as needed and takes full responsibility for the published article.

## Funding

This research did not receive any particular grant from funding agencies in the public, commercial, or not-for-profit sectors.

## Data availability statement

The authors declare that all data are available in repository.

## Declaration of competing interest

The author declares no conflicts of interest.

## References

[1] Lima Neto A.C., Mezzalira R., Bittar R.S.M. (2026). Siltation and tidal effects: a comprehensive, clinical, and didactic approach to reasoning about vertebrobasilar hypoflow conditions. Braz J Otorhinolaryngol..

[2] Gülesir M.E., Sincer İ, Gökce H.İ, Ugras U.A. (2026). Resolution of refractory vertigo following endovascular treatment of right subclavian steal syndrome. Cureus..

